# Regulation of Serum Response Factor and Adiponectin by PPAR*γ* Agonist Docosahexaenoic Acid

**DOI:** 10.1155/2011/670479

**Published:** 2010-09-19

**Authors:** Clayton Johnson, Roslyn Williams, Jeanne Y. Wei, Gouri Ranganathan

**Affiliations:** ^1^Donald W. Reynolds Institute on Aging University of Arkansas for Medical Sciences, 4301 West Markham Street, Slot no. 748, Little Rock, AR 72205, USA; ^2^Biology Department, University of Arkansas at Pine Bluff, Pine Bluff, AR 71601, USA; ^3^Central Arkansas Veterans Healthcare System, 4300 West 7th Street, Little Rock, AR 72205, USA

## Abstract

Recent studies indicate that significant health benefits involving the regulation of signaling proteins result from the consumption of omega-3 polyunsaturated fatty acids (*ω*-3 PUFAs). Serum response factor (SRF) is involved in transcriptional regulation of muscle growth and differentiation. SRF levels are increased in the aging heart muscle. It has not been examined whether SRF is made by adipocytes and whether SRF secretion by adipocytes is modulated by PPAR*γ* agonist DHA. Adiponectin is made exclusively by adipocytes. We and others have previously reported that PUFAs such as DHA increase adiponectin levels and secretion in adipocytes. Here we show that DHA downregulates SRF with a simultaneous upregulation of adiponectin and that both these responses are reversible by PPAR*γ* antagonist. Furthermore, there appears to be a direct relationship between DHA exposure and increased levels of membrane-associated high-density adiponectin, as well as lower levels of membrane-associated SRF. Thus, we find that the levels of SRF and adiponectin are inversely related in response to treatment with PPAR*γ* agonist DHA. Decreased levels of SRF along with increase in membrane-associated adiponectin could in part mediate the health benefits of DHA.

## 1. Introduction

Advancing age is associated with increased risk of cardiovascular disease, including development of metabolic syndrome, dyslipidemia, insulin resistance, hypertension, and atherosclerosis. Concurrent with the onset of these disorders there is an increase in subcutaneous and visceral adipose tissue, as well as ectopic fat within nonadipose tissues, such as liver and striated muscle (heart and skeletal muscle) [1]. This alteration in lipid distribution results in a low-grade inflammatory response caused by increases in inflammatory cytokines, such as interleukin 6 (IL-6) and tumor necrosis factor alpha (TNF-*α*), secreted by invading macrophages within the adipose depots. The adipose tissue, both subcutaneous and visceral, maintains healthy circulating free fatty acid (FFA) levels, by regulating the equilibrium between serum triglyceride and FFA levels during the fed or fasted state. 

Serum response factor (SRF) is a member of the MADS-box family of transcription factors which work in conjunction with 60 different cofactors to regulate a variety of genes in many tissues during development, maturation, and cell differentiation. Two major signaling pathways converge on SRF, the first or the classical MAPK pathway involves growth factor-stimulated phosphorylation of SRF and the second pathway involves Rho-dependent changes in actin dynamics which drives SRF transcription [2, 3]. The protein SRF is implicated in the transcriptional control of a number of cardiac muscle genes which include cardiac *α*-actin, skeletal *α*-actin, *α*-myosin heavy chain, and *β*-myosin heavy chain that may have a role in the maintenance of cardiac structure and function [4]. The cardiac muscle-specific partial SRF-knock-out mouse displays a dramatic decrease in SRF-target genes which are important to cardiomyocyte function indicating that the contractile phenotype and the calcium-handling properties of smooth muscle cells are controlled by SRF [5]. Concomitant with the age-related redistribution of adipose tissue, it has been reported that cardiac levels of serum response factor (SRF) tend to increase, and the age-associated increase in SRF appears to correlate with declines in cardiac health [6, 7]. As summarized recently by Miano [8], SRF plays a crucial role in the development and maintenance of a normal cytoskeleton. Every organ system relies on SRF for normal development and function, and clinical data indicate that levels of SRF must be strictly controlled to maintain cardiac homeostasis.

The adipose tissue is primarily an endocrine organ that secretes adipocytokines in response to changes in physiologic stimuli, thus regulating the metabolic state. One of the most abundant adipocyte-specific adipokines is adiponectin, which plays a significant role in regulating whole body insulin sensitivity, anti-inflammatory responses, and cardiac health. The adipocyte acts through the secretion of factors involved in regulating triglyceride deposition in other tissues [9]. It has been found that a modest overexpression of adiponectin in ob/ob mice resulted in increased expansion of the fat pads, relieving other tissues of triglyceride deposition and normalization of metabolic phenotype [10]. The adverse side effects of certain PPAR*γ* agonists such as thiazolidinediones that are used to manage diabetes create an impetus for identifying alternative treatments that may improve insulin sensitivity. Polyunsaturated fatty acids, which act as agonists for both PPAR*γ* and PPAR*α*, are a promising avenue for lowering of insulin resistance [11]. The long-chain *ω*-3 fatty acids, eicosapentaenoic acid (EPA), and docosahexaenoic acid (DHA), which are prevalent in certain cold-water fish oils but not in many other dietary sources, have been shown to increase the levels of plasma adiponectin in rodents by a PPAR*γ*-mediated mechanism [12]. We have recently identified that *ω*-3 fatty acids, DHA, and EPA increase adiponectin synthesis and secretion in primary cultures of rat adipocytes. Saturated fatty acid palmitate does not increase adiponectin [13]. SRF expression changes during development and adult aging, and it is suggested that preventing the age-associated elevation in SRF is associated with beneficial intracellular and physiological functional response to stress, which is advantageous for the older adult heart [7]. 

In the present paper we report that DHA regulates the level of adiponectin and SRF. We observed that the inverse regulation between adiponectin and SRF is modulated through the PPAR*γ*-mediated effects of DHA. Based on these findings, we have studied the possible correlations between *ω*-3 fatty acid (DHA) exposure and alterations in adipocyte secretion and membrane association of adiponectin as well as SRF.

## 2. Methods and Procedures

C57 BL6 mouse adipose tissues were obtained from healthy 5-6-month-old mice purchased from Harlem Sprague Dawley. Animals were maintained, and experiments were conducted according to approved protocols in accordance with IACUC guidelines. Animals were euthanized by mild CO_2_ sedation followed by cervical dislocation. Epididymal fat tissue was used to prepare adipocytes, as described previously in [14, 15]. In brief adipose tissue was minced and digested for 1 hour with 2 mg/ml collagenase in 50 mM HEPES buffer containing Krebs-ringer salts, 3% BSA, and 5 mM glucose. The digested adipocytes were separated by centrifugation at 400 × g for 1 min. Under these conditions adipocytes float and the other tissue components sediment. Adipocytes were recovered using a soft plastic pasture pipette. For experimental purposes adipocytes were incubated in DMEM/HEPES (pH 7.5) supplemented with 1% BSA, 50 U/ml of penicillin, and 5 *μ*g/ml of streptomycin and treated with BSA conjugate alone, DHA (25 *μ*M), or DHA (25 *μ*M) in the presence of PPAR-*γ* inhibitor GW9962 (1.5 *μ*M) as indicated.

### 2.1. Cell Culture and Maintenance

3T3-F442A cells were obtained from Dr. Howard Green (Harvard Medical School, Boston, MA). Cells were grown to confluence and stimulated to differentiate in DMEM (5 mM glucose) containing 10% fetal calf serum and 100 nM insulin for 14 days [16]. For experiments adipocytes were maintained in DMEM supplemented with 1% FBS or 1% BSA as specified.

### 2.2. Preparation of Fatty Acids

DHA (22 : 6, n3) (MP Biomedicals) was dissolved in DMSO and conjugated to fatty acid free BSA in a 2.5 : 1 ratio at 45°C for 30 minutes. The adipocytes were treated with 25 *μ*M DHA in presence or absence of 1.5 *μ*M of PPAR*γ* inhibitor, GW9662 (Sigma). In cells being treated with DHA the DMSO concentration of the medium was always less than 0.002%. 

### 2.3. Western Blots for SRF and Adiponectin

3T3 F442A adipocytes or mouse adipocytes were incubated in DMEM supplemented with 1% FBS, containing conjugated fatty acid for 16 h. Cells were lysed in RIPA lysis buffer containing protease inhibitor and analyzed on 10% SDS-PAGE followed by Western blotting with SRF C-terminus antibody (Santa Cruz) or adiponectin antibody (R&D systems). The SRF-C-terminus antibody detects both full-length and cleaved 32 kDa products of SRF [17]. Western bots were quantified, and data are expressed as percent of control in arbitrary units. 

### 2.4. Fractionation of Adipocyte Medium Following Treatment with DHA or DHA in the Presence of PPAR*γ* Inhibitor (GW9662)

5–20% sucrose gradients were prepared in 10 mM HEPES and 125 mM NaCl (pH 8), poured stepwise (5, 10, 15, and 20%) in 2 ml thin walled tubes and allowed to equilibrate at 4°C. Adipocyte conditioned medium is fractionated on sucrose gradients as described earlier [18]. 500 *μ*L of adipocyte-conditioned medium collected after 16 h of incubation was layered on the gradient and separated by ultracentrifugation at 310000 × g for 4 h using an SW60 Beckman rotor. Equal fractions were collected from the top of the gradient for analysis by Western blots. Equal volumes from each fraction were boiled with SDS sample buffer containing *β*-mercaptoethanol and separated on 10% SDS-PAGE. The proteins were transferred to PVDF membrane for 1 h at 250 mA and probed with antiadiponectin, Flotillin, or SRF antibody followed by HRP-conjugated secondary antibody. Antibody binding was detected by enhanced chemiluminescence (ECL) according to the manufacturer's specification (Amersham Scientific, MA).

### 2.5. Total RNA Isolation and Real Time RT-PCR

RNA isolation from primary cultures of mouse adipocytes treated with DHA and real time PCR was performed as described earlier by us [19]. Total RNA from adipose tissue was extracted using an RNeasy Lipid Tissue extraction kit from Qiagen (Valencia, CA). The quantity and quality of the isolated RNA was determined using Agilent 2100 Bio analyzer (Palo Alto, CA). Total RNA (0.5 *μ*g) was reverse transcribed using random hexamer primers with TaqMan reverse transcription reagents (Applied Biosystems, Foster City, CA). Reverse-transcribed RNA was amplified with SYBR Green PCR Master Mix (Applied Biosystems) plus 0.3 mM of gene-specific upstream and downstream primers during 35 cycles on a Rotor-Gene 3000 using Real-Time Thermal Cycler (Corbett Research, Sydney, Australia). Each cycle consisted of denaturation at 94°C for 20 s, annealing at 58°C for 20 s, and extension at 72°C for 20 s. Amplified 18S expression was used as a standard control to normalize the differences in individual samples. Primer sequences for mouse SRF and mouse adiponectin RNA are described below. Data are expressed in relation to 18S RNA. The data represent arbitrary units that accurately compare the samples to each other within that assay. The Ct values of the PCR reactions were generally between 20 and 30 for all assays. 

Mouse SRF (FR) 5′-TTGGGGCCCTCTCTCCAGCC. (RV) 5′-GGGGCCGGGCTCTTTTGACC. Mouse Adiponectin (FR) 5′-GTTGCAAGCTCTCCTGTTCC. (RV) 5′-ATCCAACCTGCACAAGTTCC. 18S (FR) 5′-TTCGAACGTCTGC-3′. (RV) 5′-ATGGTAGGCACGGCGACTA-3′.

### 2.6. Statistics

Data are expressed as mean ± SE, Student *T*-test is used for statistical analysis, level of statistical significance is set at *P* ≤ .05.

## 3. Results

### 3.1. Effect of DHA on SRF and Adiponectin Levels in 3T3-F442A Adipocytes

 Our previous studies have shown that polyunsaturated fatty acids, DHA, and EPA increased adiponectin synthesis and secretion via PPAR*γ*-mediated mechanisms in adipocytes, and saturated fatty acid palmitate at the same concentration did not alter adiponectin [13]. To determine whether DHA treatment of adipocytes regulated SRF levels via PPAR*γ*-mediated mechanisms, 3T3-F442A adipocytes were treated with 25 *μ*M DHA, 25 *μ*M DHA + 1.5 *μ*M GW9662, or GW9662 alone for 16 h. 

**Figure 1:**
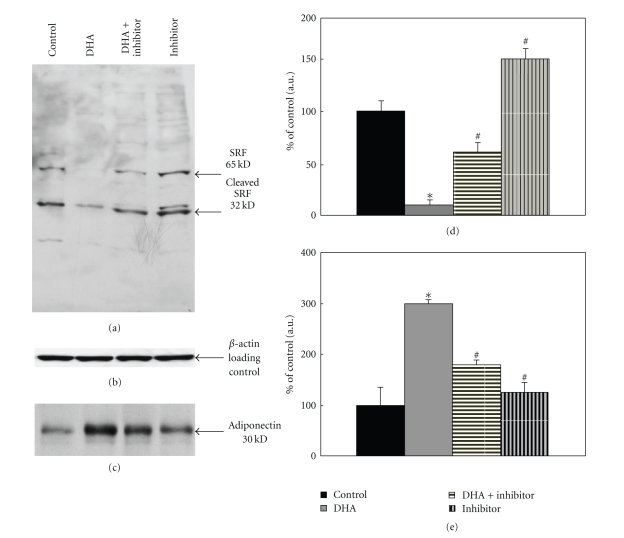
Effects of DHA on SRF and adiponectin in 3T3-F442A adipocytes. 3T3-F442A adipocytes differentiated as described in Methods were treated with 25 *μ*M DHA, 25 *μ*M DHA plus 1.5 *μ*M GW9662 or 1.5 *μ*M GW9662 in DMEM containing 1% FBS for 16 h. Cells were lysed and separated on 10% SDS-PAGE followed by Western blotting with specific antibody. (a) Blots were analyzed with SRF C-terminus antibody. (d) Bar graph shows quantification using densitometric analysis of ≈65 kDa SRF protein. Data in the bar graph represents means of three independent experiments (*P < .025, ^#^ *P* < .05). (b) The same blot was analyzed with *β*-actin antibody to examine equal loading of proteins. (c) The same samples were also examined for the expression of adiponectin. Blots were probed with adiponectin antibody; bar graph shows quantitation of adiponectin protein using densitometric analysis and is expressed as percent of control. (e) Data in the bar graph represents means of three independent experiments (*P < .05, ^#^ *P* < .05).

 Adipocyte lysates were prepared in RIPA buffer containing protease inhibitors, and 15 *μ*g protein extracts were separated on SDS-PAGE followed by analysis using Western blotting to detect SRF protein. SRF-C-terminus antibody detected ≈65 kDa full-length and ≈32 kDa cleaved SRF products as shown in Figure 1(a). Densitometric analysis of the relative expression of 65 kDa SRF is shown in the bar graph. As shown in Figure 1(a), SRF expression was inhibited by 85 ± 5% (*P* < .025) following treatment with DHA. This inhibitory effect of DHA treatment was abolished by 40 ± 5% (*P* < .05) in the presence of PPAR*γ* inhibitor GW9662, and the addition of PPAR*γ* inhibitor GW9662 to adipocytes increased SRF expression by 50% (*P* < .05). The Western blot in Figure 1(a) represents one of three similar experiments. The blot was subsequently probed with *β*-actin antibody to compare the samples for equal protein loading. There was no change in *β*-actin expression (Figure 1(b)). 

The protein extracts from 3T3-F442A adipocytes (5 *μ*g protein lysate) treated with 25 *μ*M DHA, 25 *μ*M DHA with 1.5 *μ*M GW9662, or GW9662 alone were analyzed for adiponectin expression. Adiponectin protein in adipocyte lysates was increased by 2-3-fold with DHA treatment (*P* < .05), and the addition of PPAR*γ* inhibitor along with DHA abolished the increase in adiponectin significantly (*P* < .05). PPAR*γ* inhibitor alone did not alter adiponectin significantly as compared to control (Figure 1(c)). Blots were probed with *β*-actin antibody to verify equal protein loading; there was no change in *β*-actin (data not shown). The Western blot shown represents one of three similar experiments. Bar graph represents means of all three experiments.

### 3.2. SRF Expression in Primary Cultures of Mouse Adipocytes Treated with DHA

**Figure 2:**
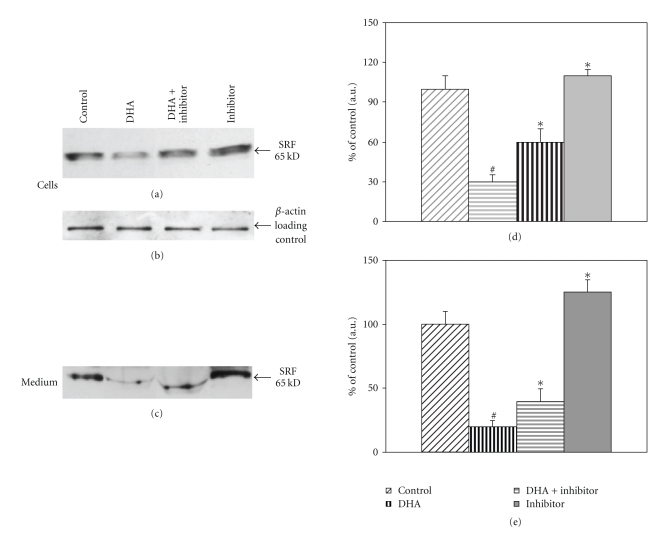
SRF expression in mouse adipocytes treated with DHA. Equal volumes (1-2 ml) of freshly isolated adipocytes were incubated in DMEM-HEPES supplemented with penicillin-streptomycin, containing 1% BSA. Adipocytes were treated as specified control, 25 *μ*M DHA, 25 *μ*M DHA plus 1.5 *μ*M GW9662, or 1.5 *μ*M GW9662 alone for 16 h. Cells and medium were separated and analyzed for SRF expression. (a) Western blots of SRF expression in mouse adipocytes. (d) Data in the adjacent bar graph represents one of two experiments done in duplicate(*P < .03, ^#^ *P* < .05). (b) The blot from part A was probed with *β*-actin antibody. (c) Western blot of SRF expression in adipocyte conditioned medium. Densitometric analysis of SRF expression in cells, and medium is expressed as a percent of control. (e) The bar graph represents means of all replicate experiments (*P < .025, ^#^ *P* < .025).

 We examined the effect of DHA on the regulation of SRF protein in freshly isolated primary cultures of mouse adipocytes. Adipocytes were prepared from epididymal fat pads of C57BL6 mice as described in Methods and incubated for 16 h in the presence of DHA (25*μ*M) or DHA + GW9662 (1.5 *μ*M) or GW9662(1.5 *μ*) alone. Cells and medium were collected separately; cells were extracted in RIPA lysis buffer containing protease inhibitors and analyzed for SRF expression using Western blots. As shown in Figure 2(a), SRF protein was inhibited by 70± 10% by DHA treatment (*P* < .025). The addition of GW9662 (1.5 *μ*M) along with DHA decreased the inhibition partially to 55 ± 10% of the control (*P* < .05), and the addition of GW9662 alone increased SRF by 10 ± 5% above the control. The same blot was probed with *β*-actin antibody to verify equal protein loading; there was no significant change in *β*-actin (Figure 2(b)). SRF secretion into the medium is inhibited by DHA treatment 80 ± 5% compared to control (*P* < .025), the addition of GW9662 along with DHA increased SRF levels to 40 ± 5% (*P* < .05), and addition of GW9662 to adipocytes increased SRF levels in the medium to 125 ± 10% of control (*P* < .05) (Figure 2(c)); Western blot shown represents one of two experiments done in triplicate. Bar graphs represent means from densitometric analysis of all replicates from two experiments. 

We examined SRF and adiponectin mRNA in primary cultures of mouse adipocytes treated with DHA, DHA+ GW9662, or GW9662 alone for 16 h. Changes in SRF and adiponectin mRNA were measured with reference to 18s RNA as described in Methods. There was no significant change in SRF mRNA or adiponectin mRNA expression (Table 1). Plasma adiponectin increased following treatment with PPAR*γ* agonist thiazolidinedione and also by treatment with *ω*-3 fatty acids [12]. However, a corresponding increase in adiponectin mRNA expression was not detected in all instances [12, 20]. In a previous study, PPAR*γ* agonist-pioglitazone increased plasma adiponectin but no corresponding increase in adiponectin mRNA was found in subcutaneous adipose tissues following pioglitazone treatment of human subjects [21]. Treatment of rat adipocytes with DHA or EPA increased cellular adiponectin protein with no corresponding increase in mRNA [13].

### 3.3. Fractionation of Medium Secreted by Adipocytes on Sucrose Gradients

 It is known that PPAR*γ* agonist increases the adiponectin secretion in adipocytes [13, 22]. To examine the coordinate regulation of adiponectin and SRF secretion by DHA, primary cultures of mouse adipocytes were treated with DHA (25 *μ*M) or DHA in the presence of PPAR*γ* antagonist, GW9662 (1.5 *μ*M). 

**Figure 3:**
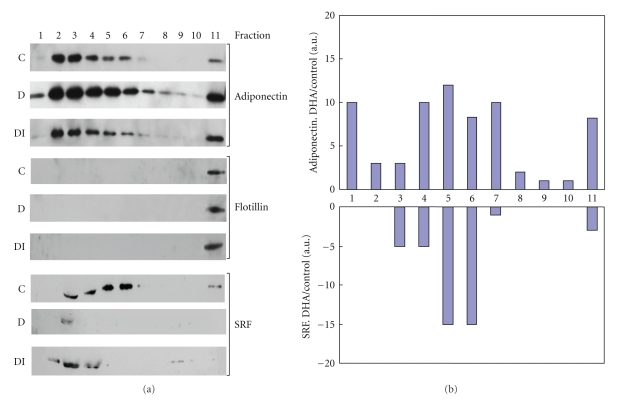
Fractionation of adipocyte medium following treatment with DHA or DHA in the presence of PPAR*γ* inhibitor GW 9662. Mouse adipocytes were treated**: **control (C), 25*μ*M DHA (D), or 25 *μ*M DHA plus 1.5 *μ*M GW9662 (DI). Equal volumes of medium secreted from treatments were fractionated on sucrose gradients and separated into 11 equal fractions as described in Methods. (a) Equal volumes of each fraction were separated on SDS-PAGE gels and analyzed by Western blotting using adiponectin, Flotillin, or SRF antibody as described in Methods. (b) Bar graph represents densitometric analysis of change in adiponectin and SRF expression following DHA treatment in each fraction: DHA/Control.

Adipocyte-conditioned medium from control-, DHA- and DHA + GW9662- treated adipocytes was centrifuged at 3000 × g to separate debris and fractionated on 5–20% sucrose gradients as described in Methods. Equal volumes (15 *μ*l) of fractionated medium from control, DHA, or DHA + GW9662 treated adipocytes were separated on 10% SDS-PAGE, and all three blots were examined simultaneously for adiponectin followed by Flotillin and SRF sequentially. Adiponectin is known to be posttranslationally modified into trimers, hexamer, and high molecular weight forms. As shown in Figure 3(a) adipocyte-conditioned medium contains adiponectin fractionating between fractions 1–7 and fraction 11 of control adipocytes, DHA treatment increased all isomeric forms of adiponectin by 2–10-folds in various fractions, as indicated by densitometric analysis of fractionated adiponectin (DHA/control) shown in Figure 3(b). The DHA-mediated increase in adiponectin release was abolished by the addition of PPAR*γ* antagonist, GW9662 (Figure 3(a)). The same blots were then simultaneously probed for the presence of Flotillin, which is a membrane marker, to assess the membrane association of adiponectin secreted by adipocytes. Flotillin was detected only in Fraction 11 of control, DHA treated, and DHA + GW9662 treated adipocytes. Fraction 11 is the densest fraction of the gradient (20% sucrose) and Flotillin expression in fraction 11 did not change with DHA treatment. 

The same blots were deprobed and probed with SRF antibody. SRF was detected in fractions 3, 4, 5, 6, and 11, in conditioned medium fractionated from control adipocytes. DHA treatment decreased SRF in fractions 3, 4, 5, 6, and 11 (Figure 3(b)) and treatment with DHA + GW9662 (PPAR*γ* antagonist) partially restored SRF levels to quantities that are detectable after fractionation on sucrose gradients (Figure 3(a)). The results in Figure 3 indicated that fraction 11 contained membrane marker Flotillin as well as both adiponectin and SRF in the conditioned medium from control adipocytes. These findings suggest that there is a probable membrane association of both adiponectin and SRF proteins.

## 4. Discussion

The molecular links between diabetes and cardiovascular disease have not been fully understood. Adiponectin is secreted by adipose tissue and is the most abundant protein in plasma, second only to albumin [23]. Plasma adiponectin levels inversely correlate with the risk for myocardial infarction, indicating that reduced adiponectin levels likely play an important role in the pathogenesis of cardiovascular disease. This hypothesis is also supported by previous studies which indicate that adiponectin knock-out mice have an increased cardiac ischemia/reperfusion injury and this injury was partially attenuated by administration of globular adiponectin [24]. SRF is known to regulate muscle growth and differentiation [25]; aging also alters the levels of SRF and factors regulated by SRF that promote muscle regeneration [26]. 

Based on the significance of both adiponectin and SRF to cardiac health in the adult and aging population, we have initiated experiments to understand the correlation between increasing the healthy *ω*-3 fatty acids or DHA and the resulting modulation of adiponectin and SRF at the cellular level. Increasing plasma adiponectin level or increasing adiponectin sensitivity with the aid of PPAR*γ* agonists has been proposed as a promising therapeutic strategy for patients with metabolic and/or cardiovascular disease [27]. Our previous studies in rat adipocytes and 3T3 F442A cells indicate that DHA increased both the biosynthesis and release of adiponectin from, adipocytes and the increase in adiponectin by DHA was blocked by PPAR*γ* antagonist [13]. Fish oil feeding for 15 days raised plasma adiponectin levels in mice, and this increase in plasma adiponectin was blocked by PPAR*γ* inhibitor [12]. 

In the present study, we observed that DHA treatment of adipocytes decreased SRF levels in both the cytosolic and secreted fractions along with increases in adiponectin. The decrease in SRF protein as well as the increase in adiponectin by DHA treatment was reversible by the addition of PPAR*γ* antagonist indicating that both these processes are likely to be PPAR*γ* mediated. This is the first report to our knowledge showing the presence of SRF in adipocytes or as a component of the adipocyte-secreted proteins. We find that DHA treatment of adipocytes decreases SRF protein levels as well as SRF secretion with no corresponding decrease in SRF mRNA expression, indicating that the decrease is mediated likely through changes in post transcriptional processing. To the best of our knowledge, the present study is the first to report the presence and secretion of SRF by adipocytes. 

Furthermore, sucrose gradient fractionation of adipocyte medium shows significant increases in the levels of all isomeric forms of the adiponectin after DHA exposure and comigration of the high density form of adiponectin with the integral membrane protein Flotillin. Our experiments also indicated that DHA treatment of adipocytes decreased the secretion of SRF and the appearance of SRF in the membrane-associated adipocyte secreted fraction. This observation indicates that the secreted high density form of adiponectin might be membraneassociated. Most of the beneficial properties of adiponectin have been ascribed to the HMW isoform which is known to have insulin sensitizing properties as well as cardioprotective functions [28, 29]. It is known that the presence of polyunsaturated fatty acids, particularly *ω*-3 fatty acids in the cell membrane, increases membrane fluidity, which results in altering the association of membranes with membrane proteins (receptors and enzymes) [30]. The adipose tissue acts as an endocrine organ by secreting the adipocytokines adiponectin, leptin, resistin, TNF-alpha, and IL6, all of which serve to divert lipid accumulation from muscle and liver to adipose tissue thus regulating insulin sensitivity and inflammation as well as cardiac health. Adiponectin, leptin, and resistin are made in significant quantities exclusively by adipocytes [9]. It is also known that plasma adiponectin concentration is decreased with ischemic heart disease and obesity and that adiponectin has strong cellular protective properties, acting through the AMPkinase- and cyclooxygenase-2-activated mechanisms [31]. Furthermore SRF is a member of a family of transcriptional activators implicated in the regulation of cell proliferation and differentiation through the regulation of the immediate early genes (IEGs) and many signaling pathways. SRF levels in the heart are increased with senescence and also in congestive heart failure [7]. Therefore it might be beneficial that fish oils decrease SRF secretion by adipocytes along with increasing the secretion of adiponectin.

## 5. Conclusions

Based on these results, we conclude that DHA, which is present in cold-water fish oils, triggers the induction of adiponectin along with reducing the levels of SRF and thereby likely mediate the desired protective downstream effects which are under investigation.

##  Disclosure

All authors attest that there are no conflicts of interest to declare.

## Figures and Tables

**Table 1:** SRF and adiponectin expression in mouse adipocytes.

Treatment	Control	DHA	DHA + GW9662	GW9662
SRF mRNA	1.442 ± 0.2	1.39 ± 0.13	1.20 ± 0.10	1.39 ± 0.2
Adiponectin mRNA	3.1 ± 0.3	2.8 ± 0.12	2.8 ± 0.3	2.9 ± 0.2

Data are mean ± SD of two experiments done in triplicate.

SRF and adiponectin are expressed relative to 18S mRNA in arbitrary units.
